# “I went in there, had a bit of an issue with those folks”: everyday challenges of heterosexual African, Caribbean and black (ACB) men in accessing HIV/AIDS services in London, Ontario

**DOI:** 10.1186/s12889-021-10321-x

**Published:** 2021-02-08

**Authors:** Roger Antabe, Irenius Konkor, Martin McIntosh, Erica Lawson, Winston Husbands, Josephine Wong, Godwin Arku, Isaac Luginaah

**Affiliations:** 1 The Department of Geography, University of Western Ontario, Social Science Centre, 1151 Richmond Street, London, Ontario N6A 5C2 Canada; 2 Regional HIV/AIDS Connections (RHAC), 30-186 King Street, London, Ontario N6A 1C7 Canada; 3 The Department of Women’s Studies and Feminist Research, University of Western Ontario, Social Science Centre, 1151 Richmond Street, London, Ontario N6A 5C2 Canada; 4 Ontario HIV Treatment Network, 1300 Yonge Street, Suite 600, Toronto, Ontario M4T 1X3 Canada; 5 Daphne Cockwell School of Nursing, Faculty of Community Services, Ryerson University, Podium Building, Room POD-481, 350 Victoria St., Toronto, Ontario M5B 2K3 Canada

**Keywords:** African, Caribbean, Black, Heterosexual, HIV, Men, Preventive health, Ontario

## Abstract

**Background:**

In Canada, heterosexual African, Caribbean, and Black (ACB) men’s heightened risk of HIV infection has been linked to behavioral characteristics, including practices of hegemonic masculinity that discourage the use of HIV preventive services. However, this framing is bereft of the role of structural factors that may be contributing to new HIV infections. This paper examined the underlying factors limiting access to health services among heterosexual ACB men in London, Ontario Canada.

**Methods:**

A convenient sampling technique was used to recruit thirty-seven (*n* = 37) self-identified heterosexual ACB men and service providers. Four focus groups (FG) were conducted; three with ACB participants of similar age category (i.e., 16–24; 25–38; 39+), and one with service providers. The FGs focused on the barriers to using health services and interrogated the ease of access to HIV intervention programs by ACB men respectively. Recurring themes from the FGs were probed further using in-depth interviews (*n* = 13). FGs and in-depth interviews complemented each other in reducing uneven power dynamics, fact checking, and allowing for detail discussion of the topic under study. Data analyses were done in NVivo using a mixed inductive-deductive thematic analyses approach.

**Results:**

Most ACB men lacked information on HIV and were unaware of their increased risk of infection. Contrary to the notion that behavioral characteristics keep ACB men away from health services, we found that most ACB men were unaware of the availability of these services. Those that had some knowledge about the services reported that they were not appropriately tailored to their needs. In addition, stereotypes and stigma about the etiology of HIV among Blacks, and systemic neglect served as significant barriers to ACB men’s use of services.

**Conclusion:**

The findings suggest that, to enhance preventive health service use among heterosexual ACB men, there is the need to remove structural barriers. Engaging ACB men in the design and implementation of policies may be useful at improving access to HIV information, testing, and treatment services. Increased information dissemination to ACB men would create awareness of the availability of HIV services. Finally, service providers should be conscious of ACB men’s concern about experiences of discrimination and racism at service centers.

## Background

In Canada, African, Caribbean, and Black (ACB) men have one of the highest HIV prevalence rates and, compared to others, maybe more vulnerable to HIV [1, 2]. While gay, bisexual, and men who have sex with men (MSM) have been identified as the most vulnerable population accounting for most HIV infections, other groups are also increasingly becoming vulnerable [3]. For instance, recent data from the Public Health Agency of Canada (PHAC) suggests that ACB people are 12.5% more likely to be infected with HIV than people in the general population. Furthermore, the ACB population represents over 25% of new HIV diagnoses, although they constitute less than 5% of Canada’s population [3]. Within the ACB community, however, heterosexual ACB men are emerging as a vulnerable group who are yet to get health policy stakeholders’ attention, including AIDS Service Organizations (ASOs) [4]. In 2009, for instance, it was reported that heterosexual ACB men accounted for 60% of ACB people infected with HIV through heterosexual exposures in Canada [5].

While earlier studies prioritized behavioral factors, including those associated with the endorsement of traditional masculinities to explain the high HIV prevalence among heterosexual ACB men [6–8], others have pointed to the overarching role of structural factors. For this study, structural factors constitute the health and social policy decisions that are beyond the control of individuals but tend to shape the quality of health and access to health care [9, 10]. The role of these factors in determining the quality of health experienced by individuals necessitate understanding the health and specifically the HIV risk of heterosexual ACB men through the social determinants of health framework [11]. For instance, race has persisted as a barrier to accessing quality health care in Canada. This barrier may extend to accessing HIV prevention and treatment services as studies maintain that the health care needs of racial minorities have not been mainstreamed into Canada’s health care policies [12].

Among ACB people in Toronto, Ontario, Gardezi and colleagues [13] found racism to inhibit them from adequately responding to their HIV health needs. In London, Ontario, heterosexual ACB men who experienced everyday discrimination were less likely to have tested for HIV [14]. Thus, though the literature acknowledges the limited use of preventive health services among ACB men, this has not been examined through the broader scope of structural and systemic risk factors that may be predisposing them to poor health outcomes [2, 5]. Hence, this study, as part of an Ontario based research -we Speak - sought to understand barriers to heterosexual ACB men’s access to HIV preventive health services with two main objectives. First, to situate heterosexual ACB men’s access to HIV-related services within broader structural factors, including social, economic, political, and environmental factors in Canada. Second, to understand how the intersection of race and sexuality inform heterosexual ACB men’s access to HIV-related services.

### Discourses on preventive health care among heterosexual ACB men

Although people of  ACB descent constitute distinct social groups with varying social experiences and interactions, we put them as one broad social category in this study. This merger is based on their reported susceptibility to HIV infection through heterosexual contacts and in line with previous studies (see [4, 14–16]). Race in this study is operationalized as a social and relational trait that informs and engenders an individual’s social positioning and interactions, including access to social services such as preventive health care. Generally, access to health care spaces such as hospitals and clinics is linked to positive and desirable health outcomes, including the acquisition of factual knowledge on disease transmission. Thus, for HIV researchers and scholars, preventive health care is useful in reducing new infections among high-risk groups such as heterosexual ACB men [17]. Additionally, increased visits and interaction with health professionals improve health literacy and help modify health behaviors [18].

Accordingly, frequent visits to health facilities provide clinicians the opportunity to test, counsel, and engage individuals and groups at high risk of HIV infection [19]. Thus, in line with its benefits to achieving the Joint United Nations Programme on HIV and AIDS (UNAIDS) 90–90-90 target by 2020, health facilities incorporate education, screening, and counseling services as part of their core preventive health care mandates [20, 21]. In these facilities, those that test positive can receive counseling on treatment options that will ensure they live their normal lives and reduce the chances of transmitting the virus [22]. Those testing negative in these facilities have the opportunity to acquire information on HIV transmission that is useful for modifying health behaviors and reducing their exposure to HIV [23].

Despite the utility of preventive health services for high-risk populations, ACB men may not use these services as frequently as recommended [14]. In explaining this phenomenon, the narrative has centered on ACB men’s behavioral characteristics that discourage their use of these services [16]. For instance, ACB men are said to be more likely to practice traditional forms of masculinity that do not only lead to the feminization of preventive health care, but may encourage risky behaviors such as partner concurrency and condomless sex that increases their exposure to HIV [4, 16]. It is further suggested that traditional masculinity’s pervasiveness in ACB communities entrenches heteronormative discourses and gender stereotypes that normalizes curative rather than preventive health care for ACB men [6, 22].

In Canada, however, structural factors have persisted as determinants of health for racial minorities, including ACB men. Despite the country’s claim to universal health coverage, poor health outcomes among Blacks have been linked to their low socioeconomic status, housing conditions, employment, and immigration status, lack of prioritization of their health needs among others [4, 24]. Particularly for recent immigrants of color, navigating structural barriers to overcome financial and economic challenges to improve health may be daunting [12]. It has been suggested by Husbands et al [2]. and Remis et al. [5] that ACB men may be disproportionally exposed to HIV infection because of structural risk factors. Therefore, to help understand ACB men’s use of preventive health services, this paper explored potential structural barriers impeding their frequent use of HIV prevention and treatment services in the context of London, Ontario.

#### Theoretical context

This study is underpinned by intersectionality theory, which posits that the intersection of people’s social and political identities tends to expose them to either privilege or experiences of marginalization [25]. In this regard, experiences of poor health outcomes for oppressed and underprivileged populations, including ACB men, can be explained by the intersections of their social and political identities [10, 26]. Propounded by Kimberle Grenshaw [25] in 1989, intersectionality asserts that the intersection of varied forms of discrimination creates a ‘new normal’ for racial minorities in ways they relate to and experience society inclusive of their health outcomes. Therefore, it is challenging to disentangle multilayered axes of oppression into individual factors. This is because these factors act in unison to predispose marginalized groups such as ACB men to increased HIV risk and poor use of health services relative to others. Conscious of the heterogeneity of the lived experiences of oppressed populations, Bowleg et al. [27] contend that intersectionality theory recognises varying realities based on race, gender, class, sexuality and how these expose people to power and privilege [28–30]. Therefore, ACB men’s poor health and use of the health care system in Canada could further be compounded by their limited power to influence health policies and push to mainstream counter-narratives about their HIV vulnerability.

According to the intersectionality theory, limiting ACB men’s HIV vulnerability solely to their behavioral attributes may be problematic. This is because it excludes persistent systemic and structural barriers in their daily encounters and how these overlap with other lived realities such as racism, stereotypes, discrimination, poverty, and general social oppression in predisposing them to HIV [24, 31–33]. For instance, Paradies et al. [34] revealed that experiences of racism from health care personnel are associated with reduced use of health services by racial minorities. Additionally, Nazroo [35] observed that racial disparities in health quality are underpinned by racism, as racial minority populations continue to face several intersecting forms of discrimination and oppression that impact their health. Therefore, to understand and proffer tested policy suggestions in addressing the HIV prevalence in ACB communities, particularly among heterosexual ACB men, it is imperative to go beyond conceptualizing vulnerability as an individual level sexual exposure, to embrace a broader scope that targets the disadvantaged position of heterosexual ACB men due to the intersection of their social and political identities.

## Methods

### Study context

London is located in the South Western part of Ontario, Canada, with an estimated population of 383,822 in 2016. The city’s population is becoming racially diverse, with data from the 2016 census estimating that people of ACB descent constitute 2.6% of the city’s population, with ACB men making up the majority (i.e., 51.3%).

Compared to other cities in the province, London has one of the highest rates of infectious diseases, including hepatitis C and HIV. For instance, although by the close of 2016, new diagnoses of HIV and hepatitis C were 4.9 and 30 persons per 100,000 respectively at the provincial level, figures recorded in the city of London were 13.5 and 39 persons per 100,000 for HIV and hepatitis C, respectively. To address these disturbing trends of infections in the city, a medical emergency was declared by The Middlesex London Health Unit, which specifically targeted injection drug users (IDUs) [36].

A 2016 report on health access in the city suggests that ethnocultural and racial minority populations experience barriers in accessing health care [37]. An earlier study also emphasized the challenges faced by members of the ACB community in the city’s health care system, compelling some to seek health care outside the city [24]. Thus, to understand the HIV threats to the ACB population in London, Ontario, Baidoobonso and colleagues [33] called for a more structurally focused approach where HIV risk and vulnerability can be situated in the social determinants of health framework.

### The weSpeak study

This study is informed by findings from an exploratory study conducted in London and Toronto dubbed ‘iSpeak’ (2011–2013), which aimed to assess how HIV-related vulnerabilities emerged among heterosexual ACB men and ways they acknowledged and engaged these vulnerabilities. Currently implemented in four cities (London, Windsor, Ottawa, and Toronto) in Ontario, this larger study sought to understand HIV vulnerability and resilience among heterosexual ACB men. The focus on heterosexual ACB men’s HIV-related health needs and challenges became necessary as evidence suggested devastating impacts of rising heterosexual HIV infection among this population. To further situate their degree of vulnerability, the research focused on access to both primary and preventive health care, particularly HIV-related services. Given the intersectional and social determinants of health perspective of the study, the research also focused on the influence of lived experiences of racism and how these informed ACB men’s access and willingness to use preventive health care services. Challenges associated with accessing social services and other systemic barriers that may be impacting their use of preventive health care services were also discussed. Data collection spanned from April 2016 to March 2017. The study received ethics approval from the University of Western Ontario non-Medical Research Ethics Board (NMREB) in 2015.

#### Participant recruitment

The study recruited heterosexual ACB men from varying ethnocultural, socioeconomic, immigration, and HIV serostatus backgrounds mostly through snowball sampling, making it difficult to have an a priori sampling frame. Aiming to recruit ACB men with varying backgrounds was to help capture the heterogeneity of their lived experiences in London, Ontario. Given that ACB people are below 3% of the city’s estimated population, initial contact with study participants was through the distribution of flyers and posted notices in spaces frequently used or visited by the target population in the city. These included Black barbering salons, churches, mosques, restaurants, and activities organized during the Black History Month. Through the leadership of Black ethnocultural groups based on country of origin in Africa or the Caribbean, members interested in the study contacted the research coordinator through email or phone for initial screening and subsequent participation. Notices were also posted at some selected hospitals and health facilities, asking interested persons to contact the research team.

Additionally, the Regional HIV/AIDS Connections (RHAC) posted notices about the study on its premises. Prospective participants who contacted the research coordinator were screened. Those who were 16 years or older, spoke English or French, lived in London, self-identified as heterosexual ACB men, and gave consent to be interviewed in either an individual in-depth or a focus group setting were recruited. For service providers who participated in the study, eligibility was based on at least five years  direct experience in providing HIV-related services to clients in their organizations. In line with our ethics protocol, all participants who met the study’s inclusion criteria signed a consent form.

### Focus groups and in-depth interviews

Focus group discussions (FG) with an average of 5 participants per group lasted between 60 — 90 min, while in-depth interviews (IDI) spanned between 45 — 60 min. With the consent of participants, all focus group discussions and in-depth interviews were audio-recorded. With the help of a field notebook, the interviewer recorded emotions and gestures that could not be audio-recorded but were deemed useful in data analysis and interpretation. Before each interview session, study participants were informed about their right not to be recorded, quoted, or participate in the research. For FGs, ACB men were put into similar age cohorts to ensure resonance, especially as discussions also aimed to capture sexual practices [38–40]. Although participants from the FGs were invited to participate in in-depth interviews, all participants who were finally selected for the in-depth interviews were not part of the focus groups.

Given the potential influence of group dynamics on an individual’s participation in focus groups, especially those that may not be used to discussing such sensitive topics within a group setting, we conducted the focus group discussions and then followed this up with in-depth interviews. The IDIs offered the opportunity for a one-on-one discussion to probe further the issues that could not be discussed in detail in a group setting. Furthermore, IDIs were deemed suitable for ACB men living with HIV as it prevented the accidental disclosure of their HIV positive status within an FG setting. Participants in both FGs and IDIs were asked to identify factors beyond their control that affected their use of preventive health care. The FG themes  that were further probed during IDIs included: the specific barriers to accessing preventive health care and treatment services, the role of service providers in accessing services, knowledge of the availability of preventive health services in communities, and how the sociopolitical construction of their HIV risk affects their use of health services. The FG for service providers centered on the ease of access to their services by heterosexual ACB men, including issues of cultural competence and their perceptions of how the organization of their services could be inhibiting or discouraging ACB men from accessing these services.

Before each interview session, participants were assured pseudonyms would be used in research publications or public presentation of study findings. Participants were also informed about their right to leave the interview whenever they wished to do so. To cover the cost of transportation to the venue for the interview, each participant was given an honorarium of CAD$ 20. The sociodemographic characteristics of the research participants are provided in Tables 1 and 2. The weSpeak study interview guide for IDIs and FGs have been published in Bryce [41] in 2018.

**Table 1:** Socio-demographic characteristics of in-depth interviews and focus group participants

Participants Characteristics	Frequency (***n*** = 30)	Percentage %
**Ethno-identity**		
African	12	40.0
Caribbean	10	33.3
Black	8	26.7
**Region of Birth**		
Africa	7	23.3
Caribbean	5	16.7
Canada	16	53.3
Other	2	6.7
**Immigration Status**		
Canadian Citizen	23	76.7
Landed immigrant/Permanent resident	4	13.3
Refugee/Protected Person Claimant	2	6.7
Other/Student Study Visa	1	3.3
**Age**		
16–24	8	26.7
25–38	12	40.0
39+	10	33.3
**Employment Status**		
Employed	12	40.0
Self-employed	6	20.0
Student/part time	7	23.3
On disability	3	10.0
Unemployed	2	6.7
**Annual Income (CAD$)**		
< 20,000	18	60.0
≥ 20,000	12	40.0
**Educational Attainment**		
High school or less	12	40.0
Some college/university/completed college/university	18	60.0
Masters or higher		
**Marital Status**		
Married or common law	10	33.3
Single (never married/divorced/separated)	20	66.7
**HIV Status**		
HIV+	3	10.0
Never tested/don’t know/HIV-	27	90.0
**Religion**		
African Traditional	1	3.3
Christian	22	73.3
Muslim	3	10.0
Other/no religion	4	13.3

**Table 2:** Socio-demographic characteristics of focus group for service providers

Participants Characteristics	Frequency (n = 7)	Percentage %
**Ethno-Racial Identity**		
African/Caribbean/Black	3	42.9
White/Caucasian	4	57.1
**Country of Birth**		
Canada	5	71.4
Africa/Caribbean	2	28.6
**Gender**		
Male	5	71.4
Female	2	28.8
**Age (years)**		
30–39	1	14.3
0–49	3	42.9
50+	3	42.9
**Professional Background**		
Medicine/Nursing/Public Health	2	28.6
Social Work/Community Services/Chaplaincy	4	57.1
Other	1	14.3
**Nature of Agency**		
AIDS Service Organization	3	42.9
Community Health Organization	1	14.3
Settlement and newcomer services	1	14.3
Health Clinic/Hospital/Public Health Agency	1	14.3
Other	1	14.3
**Type of Service**		
Medical/Dental care		
Mental Health Services/Counselling	1	14.3
HIV/AIDS & Sexual Health Support	4	57.1
Other	2	28.6
**Years at Agency**		
< 10 years	1	14.3
≥ 10 years	6	85.7
**Religion**		
Christian	6	85.7
Other/no religion	1	14.3

### Data analysis

Audio-recordings were transcribed verbatim and exported into NVivo, a qualitative data analysis software. To get a holistic understanding that also captured varied experiences affecting ACB men’s access to health care especially preventive health care, all transcripts from IDIs and FGs (including those of the service providers) were analyzed together. Based on the theoretical underpinnings of how intersecting social identities influence heterosexual ACB men’s access to health care, the authors employed a mixed inductive-deductive thematic approach to data analysis. With this approach, our theme identification process was partly driven by our theoretical perspectives of intersectionality and social determinants of health. As researchers, we were conscious of how heterosexual ACB men as a marginalized group are socially and structurally positioned to suffer poor health outcomes. The remainder of the theme identification, through the inductive lenses, was then driven by the data [42, 43]. Therefore, all four members of the weSpeak research team in London, Ontario, used this approach to identify emerging themes relating to health care access and other systemic barriers to accessing preventive health care among heterosexual ACB men. For this type of analysis, the transcripts were read through several times and thoroughly by team members to familiarize themselves with the content and emerging themes. This was followed by a line-by-line coding in reference to our specific research objectives [44, 45]. Coded transcripts were exchanged among the research team members to ensure consistency and verify if selected codes represented the identified themes. A seasoned qualitative researcher in the research institution with years of qualitative research experience went through the coded transcripts and assigned themes.

## Results

In all, thirty-seven (37) participants took part in this study, consisting of four focus groups and thirteen in-depth interviews. There was one focus group for service providers (*N* = 7) and three others for 16–24 (*N* = 5), 25–38 (N = 5), and 39+ (N = 7) age cohorts. Service providers included the Regional HIV/AIDS Connection, Middlesex-London Health Unit, London InterCommunity Health Centre (LIHC) Options Testing Clinic, and LUSO. Community services discussed the type and availability of services at their facilities, including those designed for the specific needs of heterosexual ACB men.

Although ACB men come from varying geopolitical and socio-cultural backgrounds that create different social realities, interactions, and relations that may uniquely impact ways they access preventive health services, they are put together in this study. This is in line with the similarity in their vulnerability to HIV and following the classification from PHAC. Therefore, discussions further centered on cultural competence of service delivery and the implementation of programs to attract heterosexual ACB men for HIV testing or counseling. Service providers shared their experiences in serving heterosexual ACB men and possible challenges they faced in carrying out their work, including difficulties in establishing trust and delivering culturally competent services. Additionally, service providers discussed ways they could fully engage members of the ACB community and extend available testing services. ACB men discussed HIV vulnerability issues and access to preventive health care, particularly HIV-related services.

Findings revealed the persistence of structural factors that limit heterosexual ACB men’s access to preventive health care services. Interviews with participants revealed limited awareness of HIV risks and service availability, a disconnect with health care spaces, and issues of stigma both within and outside the ACB community, which prevent heterosexual ACB men from openly discussing their vulnerability, testing for HIV, and seeking treatment. Furthermore, everyday systemic challenges hindered access to and quality of social services received by ACB men, which in/directly affected their utilization of HIV services. Emergent themes signified the existence of structural barriers that work to significantly reduce heterosexual ACB men’s visits to preventive health care spaces.

### Low awareness of HIV risks and service availability

Some heterosexual ACB men were unaware of their elevated HIV risks. This resulted from lingering misconceptions that heterosexuals may be immune to contracting HIV. For instance, given misconceptions about HIV being a ‘gay disease,’ some heterosexual ACB men presumed they had a lower risk of getting infected. A participant in a focus group remarked:

I would like to say that most times, most black men, are kind of uneducated on how HIV/AIDS is transmitted. Just because I am not a heroin junky does not mean that I won't get it from having sex with a woman. Just because I am not gay doesn't mean that I won't get it from having sex with a woman, and that's what we need to remember [FG, 39+ years]

Misconceptions owing to lack of education on HIV transmission work to reduce HIV risk perceptions among some ACB men. Another participant admitted to how he may be more vulnerable to HIV because he lacked information on HIV transmission. He stated that:

I haven't used a condom since 1998, and I have never contracted a sexually transmitted disease because I like to pick and choose who I sleep with... But I am completely ignorant of the health implications... I could be dead years ago [FG, 39+]

Some subgroups of ACB men, especially recent immigrants, are at increased risk of HIV infection as they may not be aware that HIV exists as a public health issue in their new destination of Canada. This was noted by a participant when he stated:

There are just things that they [recent immigrants] do not know. They may come from an area where there is a little bit of AIDS, but they haven't talked about it. They never really talked about it there. But then you come here [Canada], and HIV/AIDS is still here, but you are just ignorant to that fact, so you might want to have a little fun with a couple of ladies, you are an ignorant immigrant, a little ignorant [IDI, 25-38 years]

Some heterosexual ACB men who required preventive health services did not know of these services’ existence in their respective communities and London as a whole. This may imply a disconnection between institutions mandated to inform and reduce new HIV infections and ACB men. Commenting on this, a research participant stated:

I feel that not many people have access to what they need. I know there are places they give out condoms, treatments, and stuff. There are sexual clinics I have been to one before, where they will give you pills if you have contracted an STI or an STD. But not many people know about that, and when they are vulnerable and having sexual intercourse with multiple partners, then they do not know that they have it, so they are just transferring it to whoever they are having sex with [IDI, 25-38 years]

Low awareness about the availability of HIV-related health services and where to locate these services works to reduce the number of ACB men visiting these facilities to test or seek treatment for HIV. This also implies that ACB men may not be getting the needed information useful in positioning themselves to reduce their exposure to the virus. A participant noted the importance of information dissemination in reducing new infections among ACB men.

I think that what we need in terms of how we should better prepare ourselves for HIV is information. I think we touched on it already. We need the information. It could come through school, it could come through services, it could come through pamphlets, but just general awareness as my colleague said about the causes [FG, 16-24 years]

According to this participant, unlimited access to HIV information, testing, and treatment services may be indispensable to position ACB men to respond to their HIV vulnerability adequately.

### A disconnect with HIV service spaces

ACB men who were aware of the existence of HIV preventive health care services bemoaned their disconnection with these spaces. They stated that they were not represented in the staffing at these institutions. A participant commenting on the low usage of preventive health care by ACB men reported:

I think it has to be in the activity, the narrow mindedness because there are many linear thinkers in the field [ASOs]. And I think those are some of the things that need to be addressed so that they can talk to anybody. And again, in the employment, is to have a reflectiveness of the diversity [IDI, 39+ years]

Participants also reported how services are not directly targeted at them as heterosexual ACB men, although this had the potential to increase their usage of services. A participant, when asked about his experiences using HIV services remarked:

I feel they don't cater to any specific subgroup like heterosexual ACB men; I feel it’s just a general service they provide... [FG, 16-24]

While being unrepresented in staffing at ASOs makes ACB men feel alienated from these spaces, the lack of cultural competence in service delivery was also identified to reduce their visits to ASOs. The lack of culturally competent and sensitive services at ASOs was reported by a service provider (SP) as he stated:

I think from a cultural lens, I will suggest that it’s kind of walking a mile in my shoes. Right now, at our organization, for example, she talks about doing outreach, and I'm the department head of HIV, but I’m just a non-ACB guy, and she is a female. I wonder the impact of speaking to the community being not as connected. I think that if it was a strong black man presenting information, I think that might land on different ears. It might land a little differently than if it is it coming from myself or her despite our best efforts [SP, 39+ years]

These lapses at service provider sites may dissuade ACB men from testing for their HIV status or seeking treatment in these spaces. An HIV positive ACB man reported challenges he faces at provider sites when attempting to access services:

I went in there, had a bit of an issue with those folks, and I asked to get a referral to another HIV clinic in order to get the medical help that I need. They told me that they wouldn't give me a referral. I asked for a second opinion. They told me that I couldn't even get a second opinion. They told me that all I had to do was go down to some other medical spot, tell them that I have HIV and that they would take care of my needs [IDI, 39+ years]

Based on these lapses at provider sites, participants commented on how ASOs can position themselves to improve services for heterosexual ACB men. Another HIV seropositive participant remarked:

As far as ASOs are concerned, I would like to see, especially when it goes down to dealing with people that are HIV positive. You can't really call them mentors, but the people we go to for help and whatnot. I would generally like to see a good split right down the middle between heterosexual and people that are not heterosexual [IDI, 39+ years]

The quote from this participant may suggest that services in these health spaces may not be tailored towards the needs of the heterosexual population as he calls for increased representation of heterosexual people in the delivery of services.

### Stigma within and outside the community

Experiences of stigma both within and outside the ACB community, may create barriers to accessing preventive health services. Participants referred to stigma outside the community as comments and actions from non-ACB people, stereotyping them as automatic carriers of the virus. Stigma within the community was in reference to the negative discourses and blame by other ACB people around how people get infected. For some ACB people, the social environment may be hostile to people living with HIV, which affects the willingness to test or use services in ASOs. A research participant commented:

Someone raised in that type of environment [with high HIV stigma], why would they ever want to tell anyone that they contracted HIV at a point. They probably wouldn't even have been tested. And it is like so if you found yourself in a situation like that now and there are cultural stigmas around having HIV, and you realize there is nothing you can do about it, it is not so crazy to see that individuals will continue to go on and act as they normally would and that only furthers the spread of the virus [IDI, 25-38 years]

While HIV stigma directly impacts ACB men’s willingness to use preventive services, this may be heightened for those with transnational ties to Africa and the Caribbean as they may entertain fears about an HIV positive status filtering back home. A participant noted:

Someone who had experienced testing, for example, before they came to Canada knows what the situation is like in their country, say in Africa, so they will have a very different view than someone from here [Canada]. There is all this judgement. I know everybody in the community I don’t want anybody in my community to know that I'm HIV positive [SP, 39+ years]

Outside the ACB community, experiencing stigma from non-ACB persons due to stereotypes about Africans/Blacks being carriers tend to discourage testing. ACB men feel stereotyped when links are drawn between their race, sexuality, and HIV prevalence in Canada. For instance, a participant in a focus group discussion observed:

I feel like it is the stigma thing. I feel because of the susceptibility of people getting HIV in countries and continents like Africa, that same stigma is put somewhat onto us. So, it is a social norm for anyone to say HIV or AIDS you automatically think of an ACB person, which is not fair and I feel that there's also a mental stigma to it as well [FG, 16-24 years]

Other ACB men shared similar stigma experiences indicating that such stereotypes affected their social relations and interactions with non-ACB members of their community. A participant in an in-depth interview remarked:

But you know if there are general perceptions that black men are of a higher likelihood or of a higher percentage to have contracted HIV, then when I am out here in London in a small concentration of black people, the way people perceive me might be based on those statistics, based on those perceived realities. People will feel threatened to interact with me even on threat of me potentially having that virus purely from just me being black [IDI, 16-24years]

Accordingly, ACB men admitted to the realities of their higher vulnerability. However, they noted that the stigma associated with these realities also served as a barrier to using health services. In disseminating HIV information, messages are to be crafted and designed in a non-stigmatizing way to increase the reception of these messages by ACB men.

I would not be OK if someone specifically walked up to me and say, hey, here is an HIV thing because you are most likely to be susceptible... I would ask, so you are saying because I am an ACB man and more likely to be susceptible, you are just pushing it on me... I feel more comfortable receiving it in a group setting than if someone walked up to only black people and hand out pamphlets and say take this because you are susceptible to HIV [FG, 16-24]

Information dissemination that tends to put ACB men on the spot and overly emphasizing them as especially vulnerable may lead to poor reception of this information and the use of recommended services.

### Systemic policy neglect

Systemic policy neglect of the health and social needs of the ACB community was identified to be partly responsible for ACB men’s low patronage of preventive health care services. In the comment below, a service provider elucidated how the ACB community regardless of their higher vulnerability may not constitute a priority group for specific policy targeting:

I'm probably looking at it from a much more macro perspective. I’m just wondering whether the political systems, the government in essence, are putting out the kinds of money that need to be put out there to address some of the issues. Now there are many people who I always look at in terms of voting powers. Certain segments of the community don't get a lot of economic action from the government because they in terms of their voting power are fairly limited to make changes. They don't necessarily put out the kinds of funds that are needed to address some of those concerns [SP, 39+ years]

ACB men also alluded to a lack of funding for social and health programs within the ACB community that could increase awareness and access to HIV preventive health care. However, in the quote below, participants agreed that other vulnerable groups continue to receive funding for health-related programs, while others have nothing:

Just some more focused social programs directed at that (HIV programs). It is always social programs that is all that we need. The funding runs out for us in our social programs, but other communities have tons of money. [IDI, 25-38 years]

These sentiments were also shared by another participant in a focus group who believed that increased political and stakeholder attention could potentially reduce the susceptibility of the ACB community members to HIV. He stated:

From a political, economic, and environmental perspective, there has to be something done on ACB men’s higher HIV risk … .We can not have ACB men differentially distributed and then, they are automatically put at higher risk of contracting these viruses and not being able to be treated as a result of that [i.e., political, economic, and environmental factors]. More resources like what the Regional HIV/AIDS Connection does in terms of needle exchange is important. It does not even really take major economic changes in a city's budget. It takes people to be invested in and caring a little bit [IDI, 25-38].

A lack of government funding that could help inform and increase access to HIV services for heterosexual ACB men could be due to its attention to other politically mobilized groups. A service provider underscored why heterosexual ACB men’s HIV vulnerabilities might have escaped health policy attention:

In my role, we like to think that we deal with health equity. We want to think that we are targeting those populations that most need it, but the reality is that sometimes we go with the biggest, which means that we are targeting the public. Even though we have statistics that say this is a population that needs very specific targeting, but it is really only 5% of the population. So, are we going to spend our campaign dollars on 5% of the population? So, when you are making those choices again, it comes down to funding and which again comes into political. Are we spending those dollars on those vulnerable populations and often the answer is no [SP, 39+ years]

Thus, while political and health policy stakeholders, including service providers, may be aware of the heightened risk of HIV infection among heterosexual ACB men, their low numbers (i.e., less than 3% of the city’s population) as a percentage of the Canadian population may not engender political interest in their health needs.

## Discussion

As part of weSpeak, an Ontario-based study on HIV vulnerability and resilience among heterosexual ACB men, this study sought to understand the broader context of heterosexual ACB men’s access to preventive health care, specifically, HIV related services by exploring their everyday challenges in accessing these services amidst their HIV vulnerability. The findings show that although ACB men come from varying backgrounds with different identities engendering different realities, they face similar substantial everyday structural challenges in their attempts to access HIV-related health services in London. This may be contrary to earlier assertions that heterosexual ACB men’s HIV vulnerability is explained by only behavioral characteristics that work to keep them away from preventive health care services [22]. Our findings demonstrate that a multiplicity of factors – including a disconnect with health care services given the absence of culturally competent services, heightened stereotypes that stigmatize heterosexual ACB people, and systemic neglect – act as structural barriers to prevent them from easily accessing preventive health care. Furthermore, it emerged that ACB men’s limited awareness of their risk of infection is underscored by their inability to access HIV information in their communities as they tend to be alienated from ASOs. This is especially the case for recent ACB immigrants from regions with higher HIV prevalence as they may not perceive any risk of HIV infection when they arrive in Canada [33]. While members of the ACB community show urgency in their desire to get timely access to information on HIV and related services, they find themselves in a conundrum as they are disconnected from sources of HIV information as well as testing and treatment services. As observed by other researchers such as Husbands et al. [45], this revelation has worrying implications for reducing new infections as ASOs and other preventive health care facilities may not be targeting the vulnerable ACB population with the much-needed information.

Given Canada’s claim to universal health care and improved access to health information, the finding that HIV transmission misconceptions persist among the vulnerable and structurally exposed ACB population due to structural barriers in access to HIV-related information and testing services is an antithesis to this claim. However, this observation may not be too surprising as Martin and colleagues [46] allude to systemic and structural failures in adequately addressing the health vulnerabilities of racial minorities in Canada. In this sense, despite the Public Health Agency of Canada’s admonition that increasing heterosexual infection disproportionately affects the ACB community [1], heterosexual ACB men still lack sufficient awareness and readily available opportunities to test. Within the context of Canada, immense policy attention – and prioritization of MSM and IDUs – aimed at a behavioral change in the form of safer sex, increased access to HIV testing and treatment, and the introduction of needle distribution programs resulted in substantial drops in new infections among this population [47–49]. Therefore, rising heterosexual infections, which disproportionally affect ACB men calls for increased structural attention in addressing their increasing HIV vulnerability. In line with earlier studies such as Husbands et al. [2], one can argue that the intersection of ACB men’s multiple structural vulnerabilities, which is primarily underpinned by their racial ‘otherness’ inform stereotypes and narratives about a self-inflicted HIV risk and poor focus by Canada’s health policy radial.

The disconnect between heterosexual ACB men and preventive health care institutions arises due to a lack of culturally competent services and ACB representation by way of staffing in preventive health care institutions. Invariably, these concerns do not only lead to feelings of alienation from services, but ACB men are also discouraged from sharing their experiences of vulnerability with the predominantly non-ACB staff as personal health disclosures tend to occur in environments of established trust [50]. Given the increasing cultural diversity of the Canadian population, it is argued elsewhere that a corresponding diversity in the healthcare workforce will improve health access for medically underserved populations, increase research on the health needs of racial minorities while ensuring minorities are also adequately represented in future health policies [51]. According to Betancourt et al. [52], this can be achieved by recruiting minorities into health systems, providing interpreter services, and tailoring health materials in languages that are sensitive and appropriate to minority populations. Furthermore, the importance of racial representation in health spaces has been posited by Zambrana and colleagues [53] to engender feelings of empathy, cultural competence, and sensitivity for racial minorities. It has further been observed that patients’ willingness to disclose sensitive information to health professionals may be predicated on similarities in social categories, including sexual orientation and race [54]. The current underrepresentation of ACB men in staffing at ASOs amidst evidence of their rising infection may also reflect the structural complacency in understanding the needs of this vulnerable population and mainstreaming their needs into the health service delivery system. In this regard, the revelation that service providers are unable to provide culturally competent services even for ACB men living with HIV implies a non-alignment of preventive health services to specific needs of vulnerable ACB men [4].

The persistence of stigma within and outside the ACB community remains one of the greatest structural barriers in addressing HIV/AIDS in Canada [24]. Among exposed populations, including ACB people, stigma arises in the community due to misconceptions fraught with misinformation about the virus’s etiology and how it is transmitted. In Toronto, Newman et al. [55] reported similar findings as some ACB people believed contracting HIV results from moral impropriety, including risky and indiscriminate sexual adventures. Therefore, for fear of being stigmatized and ostracized, HIV positive ACB people are discouraged from openly disclosing their HIV status and may experience challenges in making the needed lifestyle adjustment with potential implications for the continuous transmission of the virus in the ACB community. In preventing accidental disclosure of their HIV serostatus to their community, particularly for those living in a transnational space, they are reluctant to seek treatment or follow a strict medication regime when they test positive. Intersected with race, the discourses of heterosexual ACB men as active transmitters of HIV in the context of North America, including Canada, as observed by Miller [56] may have distanced members of this community from actively being engaged in seeking preventive health care (see Geary [57]). Earlier findings by Loutfy et al. [58], that the intersection of race, ethnicity, and gender play an essential role in experiences of HIV-related stigma in Ontario led to the call for differently targeted approaches for specific racial minority groups, particularly ACB people in reducing HIV-related stigma, instead of a generalized approach. The authors further emphasized addressing racial and sexist discourses that in/directly expose marginalized populations to HIV vulnerability.

Despite discourses that privilege HIV vulnerability among heterosexual ACB men as behavioral, structural factors as posited by Hankivsky and Christoffersen [59], persist in preventing ACB men from accessing preventive health services. The absence of structural support for social programs in ACB communities leads to low community mobilization in sensitizing community members on their heightened risk to HIV infection. The success story in reducing substantially new HIV infections among other risk groups, including MSM and IDUs over the last two decades, calls for increased attention to rising heterosexual infections (see [60]) that disproportionally affects ACB people as a marginalized population. Given their structural disadvantage as a racial minority population, they may not have the political mobilization and lobbying prowess to adequately engage and draw policy attention to their social and health vulnerabilities reflected in their increased risk to HIV through heterosexual exposures.

## Conclusion

Findings in this study suggest that Canada’s progress in maintaining HIV prevalence below endemic levels was achieved through increased access to information and preventive health services for high-risk populations. However, racial minority groups, particularly ACB men, still report endemic HIV prevalence rates. The conundrum of higher HIV prevalence among heterosexual ACB men and limited access to information, testing, and treatment exposes a structural deficit and complacency that may be worsening the HIV vulnerability  of heterosexual ACB men. To reduce new infections among this population, there is the need for policy to shift from narratives about ACB men’s avoidance of preventive health services and instead focus on overarching structural factors predisposing them to new HIV infections.

While it is worthwhile to encourage HIV testing among this population, testing services should take an integrative approach where HIV information sessions are held prior to testing to help people acquire factual knowledge about HIV transmission. This may be particularly useful as Bond et al. [61] observed among ACB men in the US that HIV testing does not always translate into accurate knowledge of HIV transmission. In some cases, it may even be wrongly perceived as prevention. Given the commitment of heterosexual ACB men to seek HIV- related information and testing services, it is crucial to engage them through frequent community outreach. Building trust between preventive health service providers and the ACB community is essential for promoting ACB men’s access to services useful in reducing HIV prevalence in the community.

## Data Availability

The data used in this study is confidential and protected by ethics protocols of the Non-Medical Research Ethics Board of the University of Western Ontario. It can therefore not be made publicly available.

## Abbreviations

ACB: African Caribbean and Black
HIV: Human Immunodeficiency Virus
AIDS: Acquired Immunodeficiency Syndrome
PHAC: Public Health Agency of Canada
UNAIDS: United Nations Programme on HIV and AIDS
MSM: Men who Have Sex with Men
IDU: Injection Drug Users
ASO: AIDS Service Organizations
SP: Service Provider
FG: Focus Groups
IDI: In-depth Interview
